# CSF total and oligomeric α-Synuclein along with TNF-α as risk biomarkers for Parkinson’s disease: a study in *LRRK2* mutation carriers

**DOI:** 10.1186/s40035-020-00192-4

**Published:** 2020-05-06

**Authors:** Nour K. Majbour, Jan O. Aasly, Eldbjørg Hustad, Mercy A. Thomas, Nishant N. Vaikath, Naser Elkum, Wilma D. J. van de Berg, Takahiko Tokuda, Brit Mollenhauer, Henk W. Berendse, Omar M. A. El-Agnaf

**Affiliations:** 1 Neurological Disorders Research Center, Qatar Biomedical Research Institute, Hamad Bin Khalifa University, Qatar Foundation, P.O. Box 5825, Doha, Qatar; 2 Department of Neuroscience, Norwegian University of Science and Technology, (NTNU), Trondheim, Norway; 3 Department of Neurology, St. Olav’s Hospital, University Hospital of Trondheim, Trondheim, Norway; 4 Clinical Epidemiology, Sidra Medical and Research Center, Doha, Qatar; 5 Department of Anatomy and Neurosciences, Neuroscience Campus Amsterdam, VU University Medical Centre, Amsterdam, the Netherlands; 6 Department of Neurology, Research Institute for Geriatrics, Kyoto Prefectural University of Medicine, Kyoto, 602-0841 Japan; 7 Paracelsus-Elena-Klinik, Klinikstraße, Kassel, and University Medical Center Göttingen, Department of Neurology, Göttingen, Germany; 8 Department of Neurology, Amsterdam UMC, location VU University Medical Centre, Amsterdam, The Netherlands

**Keywords:** Parkinson’s disease, *LRRK2* mutation carriers, Alpha-synuclein oligomers, Biomarkers, Inflammatory markers

## Abstract

**Background:**

Asymptomatic carriers of leucine-rich repeat kinase 2 (*LRRK2*) gene mutations constitute an ideal population for discovering prodromal biomarkers of Parkinson’s disease (PD). In this study, we aim to identify CSF candidate risk biomarkers of PD in individuals with *LRRK2* mutation carriers.

**Methods:**

We measured the levels of CSF total- (t-), oligomeric (o-) and phosphorylated S129 (pS129-) α-syn, total-tau (tTau), phosphorylated threonine 181 tau (pTau), amyloid-beta 40 (Aβ-40), amyloid-beta-42 (Aβ-42) and 40 inflammatory chemokines in symptomatic (*n* = 23) and asymptomatic (*n* = 51) *LRRK2* mutation carriers, subjects with a clinical diagnosis of PD (*n* = 60) and age-matched healthy controls (*n* = 34). General linear models corrected for age and gender were performed to assess differences in CSF biomarkers between the groups. Markers that varied significantly between the groups were then analyzed using backward-elimination logistic regression analysis to identify an ideal biomarkers panel of prodromal PD.

**Results:**

Discriminant function analysis revealed low levels of CSF t-α-syn, high levels of CSF o-α-syn and TNF-α best discriminated asymptomatic *LRRK2* mutation carriers from both symptomatic PD and healthy controls. Assessing the discriminative power using receiver operating curve analysis, an area under the curve > 0.80 was generated.

**Conclusions:**

The current study suggests that CSF t-, o-α-syn and TNF-α are candidate risk biomarkers for the detection of PD at the prodromal stage. Our findings also highlight the dynamic interrelationships between CSF proteins and the importance of using a biomarkers’ panel approach for an accurate and timely diagnosis of PD.

## Background

Our understanding of the genetic basis of Parkinson’s disease (PD) has increased tremendously over the past 20 years. Mutations in the gene encoding alpha-synuclein (α-syn) were the first to be associated with genetic PD. Another monogenic causative factor in PD patients is *leucine-rich repeat kinase 2* (*LRRK2*), of which more than 100 variants have been identified [1]. Asymptomatic carriers of *LRRK2* mutations constitute an ideal population for identifying predictive biomarkers of PD for several reasons: 1) a high risk of conversion to PD, 2) dopaminergic neuronal loss demonstrated by positron emission tomography (PET) scanning, and 3) similarity of the clinical phenotype of LRRK2-associated PD to that of patients with sporadic PD (sPD). While the exact involvement of LRRK2 in PD pathogenesis remains only partially understood, converging evidence suggests a role for LRRK2 in modulating inflammation [2, 3]. As PD has been proposed to start as an inflammatory disease [4, 5], it is plausible to suggest that there may be a link between *LRRK2* mutations and inflammation.

Several research groups, including ours, have explored the potential of CSF alpha-synuclein (α-syn) forms as diagnostic or progression biomarkers for PD. Total α-syn (t-α-syn) levels were reported to be lower in PD, whereas oligomeric (o-α-syn) and phosphorylated Ser129-α-syn (pS129-α-syn) appear to be elevated [6–9]. CSF core biomarkers of Alzheimer’s disease (AD) pathology have also been widely explored in PD cases. While a drop in CSF Amyloid-beta (Aβ-42) levels have been reported in PD [10], the biomarker profile of total tau (tTau), and phosphorylated threonine 181 tau (pTau) were variable [11, 12]. More importantly, the potential of the aforementioned proteins as markers for PD at preclinical stage remains largely unexplored. Carriers of *LRRK2* mutations have an elevated risk of developing PD and they therefore represent a useful population in which to identify biomarkers of prodromal PD [13]. However, there is a paucity of data on different forms of α-syn, AD-related proteins and inflammatory biomarkers in *LRRK2* mutation carriers [14–16]. In the present study, our primary objective was to identify a panel of CSF biomarkers for the early detection of PD, preferably at the presymptomatic stage. A secondary objective was to study whether CSF levels of particular biomarkers were associated with severity of clinical symptoms of PD. Towards that end, we measured the levels of different α-syn species, AD-related proteins and 40 different inflammatory markers in CSF samples from a well-characterized Norwegian cohort of 74 subjects with *LRRK2* mutations: 23 symptomatic individuals and 51 asymptomatic mutation carriers. In parallel, we included 60 patients with sporadic (i.e. idiopathic) PD (sPD) and 43 healthy control subjects (first-degree relatives of *LRRK2* mutation carriers (Ctrl)).

## Methods

### Patient selection and CSF sampling

Patient selection criteria and the method of CSF collection were as described in previous publications [16, 17]. In total, 74 Norwegian individuals from 12 different families with *LRRK2* mutations were assessed in the current study. Twenty-three patients were clinically diagnosed with PD, whereas 51 patients were healthy, asymptomatic *LRRK2* mutation carriers when enrolled in the study. These families have been extensively described in previous reports [17–19]. In addition, 60 patients with sPD and 43 age-matched controls were recruited for this study from St. Olav’s Hospital at the University Hospital of Trondheim in Norway. The control group was composed of first-degree relatives of *LRRK2* mutation carriers who were not carrying *LRRK2* mutations. PD clinical diagnoses were made by experienced senior clinicians based on guidelines described by Gelb and colleagues [20] and disease stage was assessed according to the Hoehn and Yahr (H&Y) scale. All patients with sPD were screened and confirmed to be negative for known *LRRK2* mutations. Patients with an age at onset of ≤50 years were confirmed to be negative for known pathogenic mutations in *Parkin* and *PINK1*. All family members of LRRK2 patients were examined for clinical features of PD by movement disorder specialists and found to be asymptomatic, although a few had mild premotor signs and an increased Unified Parkinson’s Disease Rating Scale (UPDRS) score (three cases scored > 10 on UPDRS-III) [21]. The *LRRK2*-mutant PD patients were taking levodopa, and some were taking dopamine agonists and/or monoamine oxidase-B (MAO-B) inhibitors.

To collect CSF, lumbar punctures were performed in overnight fasted patients between 8 and 10 am. CSF samples were aliquoted in 1.2–1.5 ml low-binding tubes, and one vial was sent for routine laboratory analysis (i.e., white and red blood cell count, total protein and glucose levels, according to the Parkinson’s Progression Markers Initiative [PPMI] protocol), whereas the majority of the vials were frozen fewer than 15 min after collection following centrifugation at 2000 g at 4 °C then sub-aliquoted and stored at − 80 °C until further analysis. All patients provided signed informed consent, and the study was approved by the Regional Committee for Medical and Health Research Ethics.

### Measurement of alpha-synuclein species

All immunoassays used to measure the different species of α-syn were developed in-house and described in previous reports [9, 22, 23] . Briefly, to capture t- or pS129-α-syn, a 384-well ELISA microplate was coated with 0.1 or 0.5 μg/ml Syn-140 (a sheep anti-α-syn polyclonal antibody) in 200 mM NaHCO_3_, pH 9.6 (50 μl/well) by overnight incubation at 4 °C, while 0.2 μg/ml of our mouse conformation-specific antibody, Syn-O2, was used to capture o-α-syn. After incubation with 100 μl/well of blocking buffer (PBS-T containing 2.25% gelatin) for 2 h at 37 °C, 50 μl/well of the CSF samples (diluted 1:2 in artificial CSF) along with serial dilutions of recombinant human t-, pS129-or o-α-syn (50 μl) were dispensed in each well, and the plate was incubated at 37 °C for 2.5 h. After washing with PBS-T, 50 μl/well of 11D12 (a mouse anti-α-syn monoclonal antibody) [9], PS129 (a mouse anti-pS129-α-syn monoclonal antibody) [9], or FL-140 (rabbit polyclonal antibody, Santa Cruz Biotechnology, Santa Cruz, CA, USA), for measuring t-, pS129-α-syn, or o-, respectively, were added to the corresponding wells and incubated at 37 °C for 2 h. Next, the plates were washed and incubated for 2 h at 37 °C with 50 μl/well of species-appropriate secondary antibody (goat anti-rabbit IgG HRP or donkey anti-mouse IgG HRP, Jackson ImmunoResearch Laboratories Inc., USA, 1:20,000 dilution). After washing, the plates were incubated with 50 μl/well of enhanced chemiluminescent substrate (Super Signal ELISA Femto, Pierce Biotechnology, USA). The chemiluminescence, expressed in relative light units, was immediately measured using a PerkinElmer Envision multi-label plate reader (PerkinElmer, Finland). CSF samples were measured in a blinded fashion and randomized for analysis, with all LRRK2 symptomatic/asymptomatic, PD and HC samples being tested together on the same ELISA microplates. A series of internal controls was also run to check for run-to-run variations. The concentrations of α-syn species in the samples were calculated using the corresponding standard curves.

### Measurement of AD biomarkers

CSF Aβ42, Aβ40, total tau (tTau), and phosphorylated threonine 181 tau (pTau) were measured using MILLIPLEX® MAP Human Amyloid Beta Tau Magnetic Bead Panel (Luminex xMAP) run on the Bio-Plex® 3D instrument (Bio-Rad Laboratories, Hercules, CA) according to manufacturer’s instructions. The kit allows simultaneous quantification of Aβ40, Aβ42, tTau, and pTau.

### Measurement of inflammatory markers

A magnetic human chemokine bioplex assay (Bio-Rad Laboratories, Hercules, CA) was used to measure 40 chemokines from human CSF samples (6Ckine/CCL21, BCA-1/CXCL13, CTACK/CCL27, ENA-78/CXCL5, Eotaxin/CCL11, Eotaxin-2/CCL24, Eotaxin-3/CCL26, Fractalkine /CX3CL1, GCP-2/CXCL6,GM-CSF, Gro-α/CXCL1, Gro-β/CXCL2, I-309/CCL1, IFN-ϒ, IL-1β, IL-2, IL-4, IL-6, IL-8/CXCL8, IL-10, IL-16, IP-10/CXCL10, I-TAC/CXCL11, MCP-1/CCL2, MCP-2/CCL8, MCP-3/CCL7, MCP-4/CCL13, MDC/CCL22, MIF, MIG/CXCL9, MIP-1α/CCL3,MIP-1δ/CCL15, MIP-3α/CCL20,MIP-3β/CCL19, MPIF-1/CCL23, SCYB16/CXCL16, SDF-1α + β/CXCL12, TARC/CCL17, TECK/CCL25, TNF-α). Bioplex assays was run according to the manufacturer’s instructions using the recommended 1 in 2 dilution of CSF. Plates were read using the Bio-Plex® 3D suspension array system; the next generation multiplexing platform based on xMAP technology. Enclosed standards were used to generate an 8-point standard curve to which a 5-parameter logistic curve was fitted and were used to quantify unknown concentrations using BioPlex manger software. The coefficient of variance between duplicates was mostly < 10%, as was the variance between standard curves run on separate plates. Only chemokines with robust readings above background were considered for further analysis.

### Statistical analysis

IBM SPSS software (version 24.0, Chicago, IL, USA) was used for the statistical analyses of the data, whereas R 3.6.1 was used for plotting the box and whisker plots.

Demographic and clinical characteristics were compared between study groups using chi-square tests, analysis of variance with post hoc Bonferroni tests or Kruskal- Wallis tests followed by Mann-Whitney U tests, where appropriate. All datasets were tested for normality and the presence of outliers. As data were considered inappropriate for parametric analyses, Spearman rank-order correlation coefficients were used to examine correlations within the study group. For all CSF biomarkers that showed robust readings above background (i.e. α-syn species, AD markers and 18 inflammatory markers), differences between the diagnostic groups were assessed using general linear models (GLMs) corrected for age and gender with post-hoc Bonferroni corrections for multiple comparisons. Only biomarkers that varied significantly among the study groups were included in the next Discriminant Function analyses. Since our main objective was to identify predictive markers for the early detection of PD, both symptomatic *LRRK2* mutation carriers and sPD groups were combined for discriminant function analysis. Discriminant function analysis evaluates canonical discriminant functions based on combinations of the selected markers which contribute maximally to group separation and assesses how well these canonical discriminant functions discriminate the diagnostic groups. CSF biomarker data were Z-transformed prior to discriminant function analysis.

We also performed multivariate logistic regression analyses with backward stepwise selection (separate analyses for each comparison). The control group was entered as a reference category and t-, o- and pSer129-α-syn, TNF-α and IL-16 were enrolled as predictors. For the resulting models, we report AUC, sensitivity, specificity and OR (95% CI) of the individual model.

## Results

### Patient population and demographics

Demographics, clinical characteristics and CSF biomarkers levels of the study groups are summarized in Table 1. Twenty-three of the 74 subjects (30%) with *LRRK2* mutations analyzed had a manifest PD and were carrying either the most common *LRRK2* mutation, G2019S, or a different *LRRK2* mutation, N1437H. The symptomatic *LRRK2* mutation carriers had a mean age of 60 ± 11 years. 51 subjects of the 74 subjects (70%) had no symptoms of PD at the time of CSF sample collection and these cases had a mean age of 57 ± 14 years. There was no significant difference in disease duration between the symptomatic *LRRK2* mutation carriers and the sPD patients. Moreover, no difference was found between groups with regard to the routine CSF levels of white-red cell count as well as the total protein, albumin, and glucose levels, including the plasma glucose level. Controlling for age and gender did not significantly alter the results.

**Table 1:** Demographics and CSF biomarkers by diagnostic group

	Ctrl (*n* = 43)	sPD (*n* = 60)	Asymptomatic *LRRK2* mutation carriers (*n* = 51)	Symptomatic *LRRK2* mutation carriers (*n* = 23)
Age (y), mean ± SD^a^	49 ± 18	57 ± 10	57 ± 14	60 ± 11
Gender (male), n(%)^a^	19 (38.0%)	36 (52.9%)	26 (50%)	6 (25%)
Disease duration (y)^b^	NA	4 (1–6)	NA	7 (5–19)
MoCA^c^	27 (27–29)	27 (25–28)	27 (26–28)	25 (23.7–27)
H&Y	NA	2 (2–2)	0 (0–0)	2 (2–3)
UPDRS-III^d^	NA	24 (19–29)	3 (0.25–5)	24 (19.5–27)
t-α-syn (pg/mL)^e^	816 (596–1112)	573 (466–710)^***^	617 (431–803)^**^	608 (432–740)^**^
o-α-syn (pg/mL)^e^	161 (148–186)	187.5 (170.5–219.8)^**^	183 (160–230)^**^	182 (146–196)
pS129-α-syn (pg/mL)^e^	116 (103–145)	139 (114.25–163)	121 (94–150)	122 (106–145)
tTau (pg/mL)^f^	141.2 (89.6–201.3)	134.7 (88.3–224.9)	123.6 (86.7–236.4)	113.5 (94.9–167.8)
pTau (pg/mL)^f^	12 (9–16)	14.3 (9.6–21.7)	14.7 (10.7–20.8)	15.9 (10.5–22.4)
Aβ-40 (pg/mL)^f^	2506 (1967–3120)	2595.7 (2193.7–3098.2)	2776.2 (2287.6–4020.6)	3048.9 (1959.3–3810.8)
Aβ-42 (pg/mL)^f^	517.9 (404.77–738)	535.4 (405.11–662.6)	580.3 (457.8–905.3)	539.9 (410.9–880.9)
IL-16^g^	6.5 (4.9–8)	5.1 (4–6.6)^*^	5.1 (3.9–6.8)	4.3 (2.8–5.3)
TNF-α^g^	3.3 (2.6–5.5)	4.2 (2.4–6.5)	5.5 (4.2–7.4)^*^	5.5 (2.5–7.3)

Data are expressed as mean ± SD, median (IQR), or n (%). Demographical differences between groups were analyzed using analysis of variance with post hoc Bonferroni tests (age), X2 tests (gender), and Kruskal-Wallis with post hoc Mann-Whitney U tests (MoCA, H&Y, UPDRS and disease duration). Differences in CSF biomarker levels between groups were assessed with a GLM adjusted for age and gender. T-, o-, and pS129-α-syn, IL-16, TNF-α, tTau, ptau, Aβ40 and Aβ-42 were log-transformed, yet presented as raw data (* *p* < 0.05, ^**^p < 0.01, ^***^p < 0.001 compared with Ctrl)

Aβ1–42, amyloid β1–42; Ctrl, healthy controls; H&Y, Hoehn and Yahr scale; IL-16, interleukin-16; MoCA, Montreal Cognitive Assessment; NA, not applicable; o-α-syn, oligomeric α-synuclein; pSer129-α-synuclein, phosphorylated α-synuclein protein at serine 129; pTau, tau phosphorylated at threonine 181; sPD, sporadic PD; TNF-α, tumor necrosis factor-alpha; tTau, total tau protein; t-α-syn, total α-synuclein; UPDRS-III, Unified Parkinson’s Disease Rating Scale

^a^Ctrl, *n* = 43; sPD, *n* = 58; Asymptomatic *LRRK2* mutation carriers, *n* = 51, Symptomatic *LRRK2* mutation carriers, *n* = 23

^b^ Ctrl, n = NA; sPD, *n* = 59; Symptomatic *LRRK2* mutation carriers, *n* = 21

^c^ Ctrl, *n* = 15; sPD, *n* = 59; Asymptomatic *LRRK2* mutation carriers, *n* = 48, Symptomatic *LRRK2* mutation carriers, *n* = 22

^d^ Ctrl, *n* = 15; sPD, *n* = 59; Asymptomatic *LRRK2* mutation carriers, *n* = 48, Symptomatic *LRRK2 *mutation carriers, *n* = 21

^e^ Ctrl, *n* = 43; sPD, *n* = 60; Asymptomatic *LRRK2* mutation carriers, *n* = 51, Symptomatic *LRRK2* mutation carriers, *n* = 23

^f^ Ctrl, *n* = 28; sPD, *n* = 40; Asymptomatic *LRRK2* mutation carriers, *n* = 26, Symptomatic *LRRK2* mutation carriers, *n* = 11

^g^ Ctrl, *n* = 28; sPD, *n* = 42; Asymptomatic *LRRK2* mutation carriers, *n* = 24, Symptomatic *LRRK2* mutation carriers, *n* = 12

### CSF biomarkers levels in diagnostic groups

**Fig. 1:**
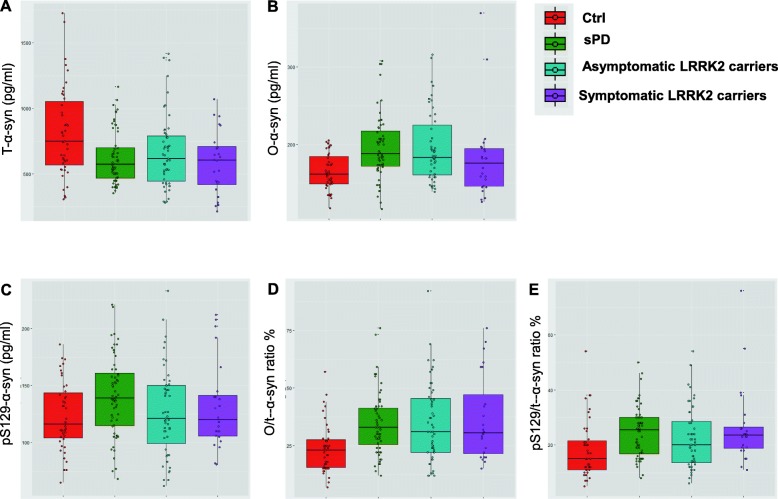
Box-and-whiskers plots of CSF levels of α-syn forms in sPD, symptomatic and asymptomatic *LRRK2* mutation carriers, and Ctrl. Box-and-whiskers plots of CSF levels of α-syn species in sPD, asymptomatic LRRK2 carriers, symptomatic LRRK2 carriers and Ctrls. **a** CSF levels of t-α-syn, **b** CSF levels of o-α-syn, **c** CSF levels of pSer129-α-syn, **d** ratio of o-α-syn/ t-α-syn %, **e** ratio of pSer129-α-syn/ t-α-syn %. The line through the middle of the boxes corresponds to the median and the lower and the upper lines to the 25th and 75th percentiles, respectively. The whiskers extend from the 5th percentile on the bottom to the 95th percentile on top. Differences between groups were assessed with the GLM compared to Ctrl group and adjusted for age and gender. *P < 0.05, **P < 0.01, ***P < 0.001

The levels of CSF t-, o- and pS129-α-syn in the different diagnostic groups are presented in Table 1 and Fig. 1. The GLM showed differences between diagnostic groups in CSF t-, o- and pS129-α-syn, TNF-α and IL-16 levels (*P* < 0.01, adjusted for age and gender; Table 1). Post-hoc tests revealed that in comparison to Ctrl (median and IQR = 816 (596–1112) pg/ml, *n* = 43), levels of CSF t-α-syn were significantly lower in sPD group (median and IQR = 573 (466.5–710.5), *n* = 60) (*P* < 0.001, Fig. 1a), asymptomatic *LRRK2* mutation carriers (median and IQR = 617 (431–803) pg/ml, *n* = 51) (*P* < 0.01, Fig. 1a), and symptomatic *LRRK2* mutation carriers (median and IQR = 608 (432–740) pg/ml, *n* = 23) (*P* < 0.01, Fig. 1a). There were no significant differences in the levels of CSF t-α-syn between the groups of sPD, symptomatic and asymptomatic *LRRK2* mutation carriers. On the other hand, and as shown in Fig. 1b, both sPD (median and IQR = 187.5 (170.5–219.8) pg/ml, *n* = 60) (*P* < 0.001, Fig. 1b) and asymptomatic LRRK2 (median and IQR = 183 (160–230) pg/ml, *n* = 51) (*P* < 0.01, Fig. 1b) groups had CSF profile with higher levels of o-α-syn compared with Ctrl (median and IQR = 161 (148–186) pg/ml, *n* = 43). Examination of CSF levels of pS129-α-syn revealed a trend of an increase in sPD group (median and IQR = 139 (114.25–163) pg/ml, n = 60) compared to Ctrl (median and IQR = 116 (103–145) pg/ml, *n* = 43), however the difference did not reach statistical significance. (Fig. 1c). The ratios of o-α-syn/t-α-syn % and pSer129-α-syn/t-α-syn were both higher in sPD, symptomatic and asymptomatic *LRRK2* mutation carriers compared with Ctrl (*P* < 0.01; Fig. 1d, e).

The age- and gender-adjusted GLM revealed no significant differences in levels of AD biomarkers between the study groups.

To determine whether inflammatory biomarkers were altered among the different study groups, a panel of 40 proinflammatory cytokines, chemokines, and growth factors were assessed in CSF samples from all study participants. Of the 40 markers measured, 18 showed robust readings above background and were further analyzed (6Ckine, IL-6, SDF-1 α + β, IL-16, MDC, MIF, TNF-α, MPIF-1, Eotaxin, IP-10, MCP-1, IL-8, MCP-2, SCYP16, MIP-1Delta, CTACK, MIP-3β, Fractalkine). GLM Bonferroni corrected analysis revealed that of the remaining 18, only IL-16 and TNF-α were significantly different. A univariate, post-hoc analysis corrected for age and gender as covariates demonstrated that IL-16 levels were significantly lower in sPD group (median and IQR = 5.1 (4–6.6) pg/ml, *n* = 42) (*P* < 0.05) compared with Ctrl (median and IQR 6.5 = (4.94–8) pg/ml, *n* = 28). More interestingly, analysis showed a significant increase of TNF-α in asymptomatic *LRRK2* mutation carriers (median and IQR = 5.5 (4.2–7.4) pg/ml (*P* < 0.05), *n* = 24) compared to Ctrl (median and IQR = 3.3 (2.6–5.5) pg/ml (*P* < 0.05), *n* = 28). The inflammatory profiles of symptomatic *LRRK2* mutation carriers and sPD cases were not significantly different (data not shown).

By use of Spearman correlations, we evaluated associations between different CSF α-syn forms and AD core biomarkers (Supplementary Table 1). For the Ctrl group, but not for any of the other groups, we found a positive association between o-α-syn and pSer129-α-syn (*r* = 0.39, *P* < 0.01). We also noted an inverse correlation between t-α-syn and o-α-syn (*r* = 0.31, *P* < 0.05) that was only present in asymptomatic *LRRK2* mutation carriers. When we explored correlations between α-syn species and the AD biomarkers, we found that t-α-syn positively correlated with Aβ-40 and Aβ-42 in the asymptomatic LRRK2 group (*r* = 0.568, *P* < 0.01, *r* = 0.485, *P* < 0.05). No other correlations were noted.

### Correlations between CSF alpha-synuclein levels and clinical parameters

Correlational analyses of CSF levels of α-syn species with clinical parameters (age, disease duration, UPDRS-III, H&Y and MoCA) are shown in Supplementary Table 2. In summary, higher levels of CSF t-α-syn correlated with: worse cognitive function as assessed by the Montreal Cognitive Assessment (MoCA) score (*r* = − 0.44, *P* < 0.01) in the sPD group. Similarly, a weak correlation of t-α-syn and age was noted in PD group (*r* = 0.29, *P* < 0.01). While pS129-α-syn positively correlated with age in PD group (*r* = 0.384, *P* < 0.01), aging was also associated with increased levels of CSF TNF-α in both PD and asymptomatic carriers groups (*r* = 0.362, *P* < 0.01, *r* = 0.492, *P* < 0.01, respectively).

### Discriminant function analysis

**Fig. 2:**
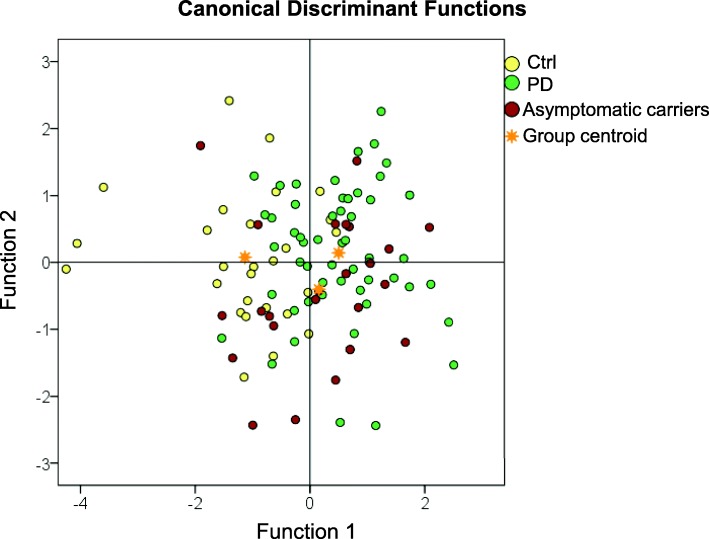
Discriminant function plot of canonical discriminant functions. CSF biomarkers were z-transformed before analyses. Discriminant function plot of canonical discriminant functions for discrimination of asymptomatic *LRRK2* mutation carriers, PD and controls. Yellow dots indicate individual data of control subjects, green dots indicate individual data of Parkinson’s disease patients (sporadic and symptomatic *LRRK2* mutation carriers) and red dots indicate individual data of LRRK2 asymptomatic carriers. The golden stars represent the group centroids

In an attempt to identify the optimal panel that can serve as predictive markers for PD at the prodromal stage, we performed a discriminant function analysis of biomarkers that were significantly different between the groups. Canonical discriminant function classification results are presented in Table 2. In the analysis, both groups of sPD and symptomatic LRRK2 carriers were combined as one PD group. A panel of t-, o- and pS129-α-syn, TNF-α and IL-16 together correctly classified 60% of all cases in the asymptomatic *LRRK2* mutation carriers, PD, and Ctrl groups (lambda = 0.644, *P* < 0.001). The discrimination plot of the two canonical discriminant functions for discrimination of the three groups is presented in Fig. 2 and the loadings of individual predictors on each discriminant function are shown in Supplementary Table 3. Canonical discriminant function 1 strongly correlated with t-α-syn (*r* = − 0.694^*^), o-α-syn (*r* = − 0.499*) and pS129-α-syn (*r* = 0.390*) and discriminated both asymptomatic *LRRK2* mutation carriers and PD groups from Ctrl group; we will subsequently refer to this function as the *Disease* function. Canonical discriminant function 2 strongly correlated with TNF-α (*r* = 0.678*) and IL-16 (*r* = − 0.554*) and further discriminated asymptomatic *LRRK2* mutation carriers group from Ctrl group; we will subsequently refer to this function as the *Prodromal* function. Asymptomatic *LRRK2* mutation carriers’ centroid is located at the intersection of both the *Disease* axis and the *Prodromal* disorders axis.

**Table 2:** Canonical Discriminant functions classification results ^a^

Group			Predicted Group Membership			Total
			ctrl	PD	Asymptomatic carriers	
Original	Count	Ctrl	20	3	4	27
		PD	7	33	14	54
		Asymptomatic carriers	5	9	10	24
	%	Ctrl	**74.1**	11.1	14.8	100.0
		PD	13.0	**61.1**	25.9	100.0
		Asymptomatic carriers	20.8	37.5	**41.7**	100.0

^a^ 60.0% of original grouped cases correctly classified

**Fig. 3:**
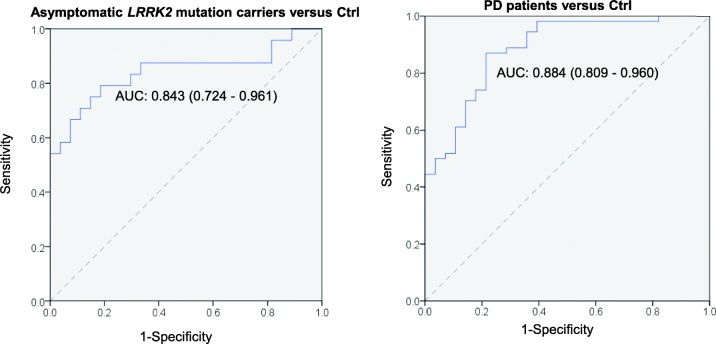
Receiver operating characteristic curves (ROC) showing the diagnostic accuracy of the final logistic regression models. Asymptomatic *LRRK2* mutation carriers vs Ctrl subjects, (B) PD patients vs Ctrl subjects

We employed backward-elimination multiple logistic regression analyses to identify optimal biomarker panels for bilateral comparisons between: [1] asymptomatic *LRRK2* mutation carriers and Ctrl, and [2] PD patients and Ctrl, for which t-, o- and pS129-α-syn, TNF-α, and IL-16 were entered as predictors and the Ctrl group was used as the reference group in each comparison. A summary of the final models are shown in Table 3. The combination of t-α-syn, o-α-syn, and TNF-α discriminated the asymptomatic *LRRK2* mutation carriers group from the Ctrl group: low levels of t-α-syn (OR, 0.997; 95% CI, 0.994–0.999), high levels of o-α-syn (OR, 1.029, 95% CI, 0.999–1.060), and high levels of TNF-α (OR, 1.418; 95% CI, 0.998–2.014) indicate that individuals are at higher risk developing PD. Examining PD and Ctrl groups only, we found that low levels of t-α-syn (OR, 0.996; 95% CI, 0.994–0.999), high levels of o-α-syn (OR, 1.031; 95% CI,1.005–1.056), high levels of pS129-α-syn (OR, 1.035; 95% CI, 1.010–1.059), low levels of IL-16 (OR, 0.785; 95% CI, 0.603–1.022) differentiates the PD group from the Ctrl group. Receiver operating characteristic curves for both models are illustrated in Fig. 3. Each of the models generated an area under the curve (AUC) of > 0.80.

**Table 3:** Logistic regression analysis of multiple CSF biomarkers

Ctrl Group				
	Predictors	OR (95% CI)	Accuracy of model	*P*
Asymptomatic carriers	t-α-syn o-α-syn TNF-α	0.997 (0.994–0.999) 1.029 (0.999–1.060) 1.418 (0.998–2.014)	AUC: 0.843 (0.724–0.961) Sens: 87.5%, Spec: 66.7%	0.000
PD (i.e. sPD & symptomatic *LRRK2* mutation carriers)	t-α-syn o-α-syn pS129-α-syn IL-16	0.996 (0.994–0.999) 1.031 (1.005–1.056) 1.035 (1.010–1.059) 0.785 (0.60–1.022)	AUC: 0.896 (0.823–0.969) Sens: 87.0% Spec: 78.6%	0.000

*AUC* area under the curve, *HC* Healthy controls, *NPV* negative predictive value, *OR* odds ratio, *o-α-syn* oligomeric α-synuclein, *PD* sporadic and symptomatic *LRRK2* mutation patients, *PPV* positive predictive value; pSer129-α-synuclein, phosphorylated α-synuclein protein at serine 129, *Sens* sensitivity, *Spec* specificity, *TNF-α* tumor necrosis factor-alpha, *t-α-syn* total α-synuclein

## Discussion

*LRRK2* mutations are well-described as a cause of genetic familial parkinsonism, and drugs inhibiting LRRK2 kinase activity are already in clinical trials [24]. However, as *LRRK2* mutation carriers are at high risk of developing PD, asymptomatic individuals with *LRRK2* mutations are an excellent group for discovery of biomarkers of prodromal PD based on the premise that they are highly likely to develop PD in future.

In the current study, we measured 47 different candidate biomarkers and found evidence that low levels of CSF t-α-syn, and high levels of CSF o-α-syn and TNF-α potentially differentiate asymptomatic *LRRK2* mutation carriers, i.e. PD subjects at the prodromal phase, from healthy controls. As these biomarkers in combination had greater discriminant power than those in isolation, these findings emphasize the value of combining multiple markers for early detection of PD.

In line with previous cross-sectional studies assessing the diagnostic power of CSF α-syn forms [8, 9], we report that sPD patients had significantly decreased levels of CSF t-α-syn and increased levels of CSF o- and pS129-α-syn compared to Ctrl group. Importantly, our results also show that asymptomatic *LRRK2* mutation carriers displayed significantly higher CSF o-α-syn than Ctrls. These findings support the hypothesis that α-syn oligomerization is an early event in the pathophysiology of PD and add further weight to evaluation of CSF o-α-syn as a candidate biomarker for detection of prodromal PD. A previous study showed a similar trend, although the results were not significant [16]. One possible explanation for disparate results between both studies is that the use of conformation-specific antibodies and oligomeric-specific ELISA in the present study, minimized the overlap between groups and improved the discriminant power between the study groups.

Our results partially contradict with Vilas et al.*,* 2016 study, where CSF t-α-syn levels were lowered only in idiopathic (i.e. sporadic) PD group in contrast to other diagnostic groups; however, the differences in CSF tTau, pTau, Aβ-40 or Aβ-42 levels didn’t reach statistical significance. Immunoassays employed to measure AD biomarkers in both studies used Luminex xMAP but employed different antibodies, which may underlie different results. If this is the case, this would have important implications for the design of future biomarkers studies.

Several biomarkers and brain tissue studies have shown that neuroinflammatory process precedes neurodegeneration in PD [25–27], whilst very few studies have evaluated inflammatory biomarkers in relation to α-syn forms [15]. In the current study, we found CSF TNF-α to be significantly higher in asymptomatic *LRRK2* mutation carriers compared to Ctrls. In a recent 11-year study in over 4 million Norwegians, an inhaled asthma medication with anti-TNF activity, a brain-penetrant drug, was associated with lower levels of t-α-syn and decreased the risk of PD [28, 29]. While the authors of the study didn’t claim a causative association, their findings correspond with our present findings, as asymptomatic *LRRK2* mutation carriers showed significantly lower CSF t-α-syn and higher TNF-α and o-α-syn. However, the control subjects in our study were significantly younger than sPD, symptomatic and asymptomatic *LRRK2* mutation carriers (*p* < 0.05), a notable finding given that previous studies have demonstrated an age-dependent increase in CSF TNF-α levels [30], which we also observed (Supplementary Table 2). However, CSF TNF-α levels were still significantly increased in asymptomatic *LRRK2* mutation carriers compared with Ctrl subjects when CSF TNF-α values were analyzed after age adjustment (Table 1). We observed no effect of gender on biomarker levels in all study groups, and analysis was performed with and without gender adjustment with no difference in overall findings. Therefore, we concluded that gender bias did not affect the results in this study.

It is important to note that *LRRK2* mutations (G2019S, N1437H) have incomplete penetrance, meaning that not all of the asymptomatic *LRRK2* mutation carriers in our cohort would develop PD. This may explain the overlap between asymptomatic *LRRK2* mutation carriers and both Ctrl and sPD groups (Fig. 1), falling almost at the intersection in the discriminant function analysis (Fig. 3). In light of the incomplete penetrance of *LRRK2*, discriminant function analysis correctly classified 60.0% of original grouped cases, where 74.1% Ctrl, 61.1% PD and 41.7% cases of asymptomatic *LRRK2* mutation carriers were correctly classified (Table 2). It is tempting to speculate that the lower discriminant function analysis levels for asymptomatic *LRRK2* cases reflects the incomplete penetrance of this mutation, and could suggest that the cases correctly discriminated are those most likely to develop PD. Future studies could further explore this question in *LRRK2* patients in large prospective studies, such as the ongoing Parkinson’s progression markers initiative, to determine their diagnostic utility in predicting PD in *LRRK2* mutation carriers.

In a recent study by Halliday and colleagues, using CSF and serum samples from the Michael J. Fox Foundation LRRK2 cohort consortium [15], 28 cytokines were measured in CSF and compared between Ctrl (*n* = 22) and asymptomatic *LRRK2* mutation carriers (*n* = 25). However, in this study, none of the markers, including TNF-α, distinguished between the two groups but CSF TNF-α levels combined with 5 other cytokines significantly differentiated these cases from sPD (*n* = 29) and symptomatic *LRRK2* mutation (G2019S) carriers. The discrepancy seen in our results and the above mentioned study could be due to the differences in the inclusion and exclusion criteria of the subjects, and/or time difference in processing or storing the samples.

Admittedly, the size of our cohort is relatively small, particularly considering the number of subjects in which all the biomarkers’ measurements were available. Furthermore, the only control group in this study was composed of first-degree relatives of *LRRK2* mutations carriers and, although they were confirmed negative for *LRRK2* mutations, including another standard control group shall be considered in further studies. Previous studies have described heterogeneous pathologies in *LRRK2* mutation carriers, where different brain areas and methods were studied and used [31–33]. Such differences in neuropathological changes elicited by *LRRK2* mutations necessitates the need for cohorts where neuropathological examination of *LRRK2* mutation carriers is conducted both to provide neuropathological confirmation of clinical diagnoses, and to better define and validate differential profiles of biomarkers.

## Conclusions

Our study, by demonstrating lower CSF t-α-syn levels, higher o-α-syn and TNF-α in asymptomatic *LRRK2* mutation carriers highlights the power of those biomarkers at providing an early detection of PD. Future studies are necessary to confirm the potential sensitivity and specificity of combing α-syn species with inflammatory biomarkers as predictive and perhaps progression biomarkers of PD. Comparison of CSF and blood levels of the current biomarkers in larger cohorts with longitudinal follow-up of *LRRK2* mutation carriers, and other “at risk” groups, are of great importance. Such studies are crucial to select biomarkers that could identify individuals at high risk to convert to PD, who would then be the target group for the development of preventive treatments.

## Supplementary information

**Additional file 1: Supplementary Table 1.** Associations between CSF biomarkers. No correlation between CSF biomarkers were present in symptomatic *LRRK2* mutation carriers group alone. Both sPD and symptomatic *LRRK2* mutation carriers groups were combined as one PD group. Associations between CSF biomarkers were assessed with Spearman correlation coefficients. Data shown as r. Significance: *** *p* < 0.001; ** *p* < 0.01; * *p* < 0.05 Aβ1–42, amyloid β1–42; Ctrl, Healthy controls; o-α-syn, oligomeric α-synuclein; pSer129-α-synuclein, phosphorylated α-synuclein protein at serine 129; pTau, tau phosphorylated at threonine 181; PD, Parkinson’s disease patients; tTau, total tau protein; and t-α-syn, total α-synuclein. **Supplementary Table 2.** Associations between CSF α-syn species and clinical parameters. Associations between CSF biomarkers were assessed with Spearman correlation coefficients. Data shown as r. Significance: *** *p* < 0.001; ** *p* < 0.01; * *p* < 0.05. Both sPD and symptomatic *LRRK2* mutation carriers groups were combined as one PD group. Aβ1–42, amyloid β1–42; Ctrl, healthy controls; H&Y, Hoehn and Yahr scale; MoCA, Montreal Cognitive Assessment; NA, not applicable; o-α-syn, oligomeric α-synuclein; pSer129-α-synuclein, phosphorylated α-synuclein protein at serine 129; pTau, tau phosphorylated at threonine 181; sPD, sporadic PD; TNF-α, tumor necrosis factor-alpha; tTau, total tau protein; t-α-syn, total α-synuclein; UPDRS-III, Unified Parkinson’s Disease Rating Scale. **Supplementary Table 3.** Discriminant loadings for each individual predictor. The correlation coefficient represents the relative contribution for each predictor to group separation. IL-16, interlukin-16; o-α-syn, α-synuclein oligomers; pS129-α-syn, phosphorylated Ser 129 α-synuclein; t-α-syn, total α-synuclein; TNF- α, tumor necrosis factor- α.

## Data Availability

The datasets used and/or analyzed during the current study are available from the corresponding author on reasonable request.

## Abbreviations

PD: Parkinson’s disease
CSF: Cerebrospinal fluid
TNF-α: Tumor necrosis factor-alpha
AUC: Area under the curve
PET: Positron emission tomography
AD: Alzheimer’s disease
UPDRS: Unified Parkinson’s disease rating scale
MAO: Monoamine oxidase inhibitor
PPMI: Parkinson’s Progression Markers Initiative
ELISA: Enzyme Linked Immunosorbent Assay
PBS: Phosphate-buffered saline
PBS-T: Phosphate-buffered saline with tween-20
HRP: Horseradish peroxidase
HC: Healthy control
OR: Odds ratio
CI: Confidence interval
GLM: General linear model
IL: Interleukin
IQR: Interquartile range
NPRP: National Priorities Research Program
