# Supplementary material for: CSF total and oligomeric α-Synuclein along with TNF-α as risk biomarkers for Parkinson’s disease: a study in LRRK2 mutation carriers
Source: Transl Neurodegener. 2020 May 6;9:15. doi: 10.1186/s40035-020-00192-4 (PMC7201744; doi:10.1186/s40035-020-00192-4)
Supplement: Supplementary file 1 — Additional file 1: Supplementary Table 1. Associations between CSF biomarkers. No correlation between CSF biomarkers were present in symptomatic LRRK2 mutation carriers group alone. Both sPD and symptomatic LRRK2 mutation carriers groups were combined as one PD group. Associations between CSF biomarkers were assessed with Spearman correlation coefficients. Data shown as r. Significance: *** p < 0.001; ** p < 0.01; * p < 0.05 Aβ1–42, amyloid β1–42; Ctrl, Healthy controls; o-α-syn, oligomeric α-synuclein; pSer129-α-synuclein, phosphorylated α-synuclein protein at serine 129; pTau, tau phosphorylated at threonine 181; PD, Parkinson’s disease patients; tTau, total tau protein; and t-α-syn, total α-synuclein. Supplementary Table 2. Associations between CSF α-syn species and clinical parameters. Associations between CSF biomarkers were assessed with Spearman correlation coefficients. Data shown as r. Significance: *** p < 0.001; ** p < 0.01; * p < 0.05. Both sPD and symptomatic LRRK2 mutation carriers groups were combined as one PD group. Aβ1–42, amyloid β1–42; Ctrl, healthy controls; H&Y, Hoehn and Yahr scale; MoCA, Montreal Cognitive Assessment; NA, not applicable; o-α-syn, oligomeric α-synuclein; pSer129-α-synuclein, phosphorylated α-synuclein protein at serine 129; pTau, tau phosphorylated at threonine 181; sPD, sporadic PD; TNF-α, tumor necrosis factor-alpha; tTau, total tau protein; t-α-syn, total α-synuclein; UPDRS-III, Unified Parkinson’s Disease Rating Scale. Supplementary Table 3. Discriminant loadings for each individual predictor. The correlation coefficient represents the relative contribution for each predictor to group separation. IL-16, interlukin-16; o-α-syn, α-synuclein oligomers; pS129-α-syn, phosphorylated Ser 129 α-synuclein; t-α-syn, total α-synuclein; TNF- α, tumor necrosis factor- α. [file 40035_2020_192_MOESM1_ESM.docx]

| Supplementary Table 1. Associations between CSF biomarkers | | | | | | | |
| --- | --- | --- | --- | --- | --- | --- | --- |
|  | t-α-syn | o-α-syn | pS129-α-syn | Aβ-40 | Aβ-42 | tTau | pTau |
| **t-α-syn** | | | | | | | |
| Ctrl | 1.000 | 0.276 | -0.078 | 0.194 | 0.125 | 0.055 | 0.234 |
| PD | 1.000 | -0.025 | 0.130 | 0.005 | -0.028 | -0.119 | -0.083 |
| Asymptomatic carriers | 1.000 | -.318^*^ | -0.048 | 0.568^**^ | 0.485^*^ | 0.225 | 0.299 |
| **o-α-syn** | | | | | | | |
| Ctrl | 0.276 | 1.000 | 0.389^**^ | 0.056 | 0.082 | 0.186 | 0.168 |
| PD | -0.025 | 1.000 | -0.140 | -0.093 | -0.084 | -0.042 | -0.247 |
| Asymptomatic carriers | -0.318^*^ | 1.000 | 0.001 | -0.152 | -0.320 | -0.035 | -0.394 |
| **pS129-α-syn** | | | | | | | |
| Ctrl | -0.078 | .389^**^ | 1.000 | 0.058 | 0.097 | 0.095 | 0.110 |
| PD | 0.130 | -0.140 | 1.000 | -0.097 | -0.184 | 0.010 | 0.014 |
| Asymptomatic carriers | -0.048 | 0.001 | 1.000 | 0.058 | -0.027 | -0.313 | -0.207 |
| No correlation between CSF biomarkers were present in symptomatic *LRRK2* mutation carriers group alone. Both sPD and symptomatic LRRK2 mutation carriers groups were combined as one PD group. Associations between CSF biomarkers were assessed with Spearman correlation coefficients. Data shown as r. Significance: *** p<0.001; ** p<0.01; * p<0.05  Aβ1-42, amyloid β1-42; Ctrl, Healthy controls; o-α-syn, oligomeric α-synuclein; pSer129-α-synuclein, phosphorylated α-synuclein protein at serine 129; pTau, tau phosphorylated at threonine 181; PD, Parkinson’s disease patients; tTau, total tau protein; and t-α-syn, total α-synuclein. | | | | | | | |

| Supplementary Table 2. Associations between CSF α-syn species and clinical parameters | | | | | |
| --- | --- | --- | --- | --- | --- |
|  | Age | UPDRS-III | H&Y | MoCA | Disease duration |
| **t-α-syn** | | | | | |
| PD | 0.290^**^ | 0.218 | 0.197 | -0.444^**^ | 0.166 |
| Asymptomatic carriers | 0.225 | -0.017 | -0.323^*^ | -0.242 | NA |
| **o-α-syn** | | | | | |
| PD | 0.025 | 0.113 | -0.061 | 0.042 | -0.114 |
| Asymptomatic carriers | -0.111 | -0.094 | 0.154 | 0.230 | NA |
| **pS129-α-syn** | | | | | |
| PD | 0.160 | 0.190 | 0.125 | -0.087 | 0.130 |
| Asymptomatic carriers | 0.384^**^ | 0.252 | 0.163 | -0.348^*^ | NA |
| **TNF-α** |  |  |  |  |  |
| PD | 0.362^**^ | 0.038 | .322^*^ | -0.178 | -0.113 |
| Asymptomatic carriers | 0.492^*^ | 0.086 | 0.383 | -0.242 | NA |
| Associations between CSF biomarkers were assessed with Spearman correlation coefficients. Data shown as r. Significance: *** p<0.001; ** p<0.01; * p<0.05. Both sPD and symptomatic LRRK2 mutation carriers groups were combined as one PD group.  Aβ1-42, amyloid β1-42; Ctrl, healthy controls; H&Y, Hoehn and Yahr scale; MoCA, Montreal Cognitive Assessment; NA, not applicable; o-α-syn, oligomeric α-synuclein; pSer129-α-synuclein, phosphorylated α-synuclein protein at serine 129; pTau, tau phosphorylated at threonine 181; sPD, sporadic PD; TNF-α, tumor necrosis factor-alpha; tTau, total tau protein; t-α-syn, total α-synuclein; UPDRS-III, Unified Parkinson's Disease Rating Scale. | | | | | |

| Supplementary Table 3. Discriminant loadings for each individual predictor | | |
| --- | --- | --- |
|  | Function | |
|  | 1 | 2 |
| T-α-syn | -0.694**^*^** | 0.326 |
| O-α-syn | -0.499**^*^** | 0.032 |
| pS129-α-syn | 0.390**^*^** | -0.131 |
| TNF-α | 0.420 | 0.678**^*^** |
| IL-16 | 0.295 | -0.554**^*^** |
| The correlation coefficient represents the relative contribution for each predictor to group separation.  IL-16, interlukin-16; o-α-syn, α-synuclein oligomers; pS129-α-syn, phosphorylated Ser 129 α-synuclein; t-α-syn, total α-synuclein; TNF- α, tumor necrosis factor- α. | | |
